# COVID-19 is associated with the risk of cardiovascular disease death: A two-sample Mendelian randomization study

**DOI:** 10.3389/fcvm.2022.974944

**Published:** 2022-09-06

**Authors:** Jia-peng Miao, Xiao-yu Gu, Rui-zheng Shi

**Affiliations:** ^1^Department of Cardiovascular Medicine, Xiangya Hospital, Central South University, Changsha, China; ^2^Department of Dermatology, Xiangya Hospital, Central South University, Changsha, China

**Keywords:** COVID-19, cardiovascular disease death, Mendelian randomization, GWAS, SNP

## Abstract

**Objective:**

This study aimed to estimate the causal effects of Coronavirus disease 2019 susceptibility and hospitalization on cardiovascular disease death using two-sample Mendelian randomization analysis.

**Methods:**

We used statistics from a genome-wide association study. A total of 2,568,698 participants were assessed in this study, including 1,299,010 in Coronavirus disease 2019 susceptibility databases, 908,494 in Coronavirus disease 2019 hospitalization database, and 361,194 in a cardiovascular disease death database. We performed two-sample Mendelian randomization analysis using the inverse variance weighted method. As sensitivity analysis techniques, Mendelian randomization-Egger regression, heterogeneity analyses, and Leave-one-out analysis were employed. Reverse Mendelian randomization analysis was used to detect reverse causality. Statistical significance was defined as *P* < 0.05.

**Results:**

Coronavirus disease 2019 susceptibility may be a causal factor for cardiovascular disease death (β = 2.188 × 10^–3^, *P* = 0.002), which involves five common single nucleotide polymorphisms. Similarly, Coronavirus disease 2019 hospitalization may also be a causal factor for cardiovascular disease death (β = 8.626 × 10^–4^, *P* = 0.010), which involves nine common single nucleotide polymorphisms. Furthermore, sensitivity and reverse Mendelian randomization analysis suggested that no heterogeneity, horizontal pleiotropy or reverse causality was found between Coronavirus disease 2019 and cardiovascular disease death.

**Conclusion:**

Our bidirectional Mendelian randomization analysis showed a causal relationship between Coronavirus disease 2019 susceptibility and hospitalization associated with an increased risk of cardiovascular disease death.

## Introduction

Coronavirus disease 2019 (COVID-19), caused by the severe acute respiratory syndrome coronavirus 2 (SARS-CoV-2), is an ongoing worldwide pandemic. COVID-19-induced clinical manifestations range from asymptomatic latent infection to severe pneumonia with respiratory failure, multi-organ failure, and rapid death (1, 2). The risk of cardiovascular disease (CVD) death increased significantly in hospitalized patients with COVID-19 (3). Patients with pre-existing cardiovascular disease and associated cardiovascular complications suffer from more severe symptoms after COVID infection. Myocardial injury accounts for approximately 25% of critically ill COVID-19 patients (4), and manifestes in the form of acute exacerbation and decompensated of chronic heart, and acute cardiovascular complications (5). However, whether there is a direct causal relationship between COVID-19 and the severity of cardiovascular disease death is unclear.

Mendelian randomization (MR) is an approach that uses genetic variation to estimate the causal relationship between risk factors and disease outcomes (6). The principle is similar to a randomized controlled trials (RCT), in which participants are randomly assigned. Furthermore, in MR, genetic variation as an instrumental variable (IV) is randomly assigned through Mendel’s second law (7). Therefore, as data from genome-wide association studies (GWAS) are gradually becoming available to the public, MR studies can serve as an alternative to RCTs to some extent, subject to ethical, technical, financial and feasibility constraints.

Our study evaluated the association between COVID-19 susceptibility and CVD death, as well as the relationship between hospitalization and cardiovascular disease death by using the GWAS database and performing two-sample MR analysis based on MR guidelines (8, 9). Two distinct dimensions of susceptibility to COVID-19 and hospitalization were employed to anayse the disease’s impact on CVD death.

## Method

### Study design

**FIGURE 1:**
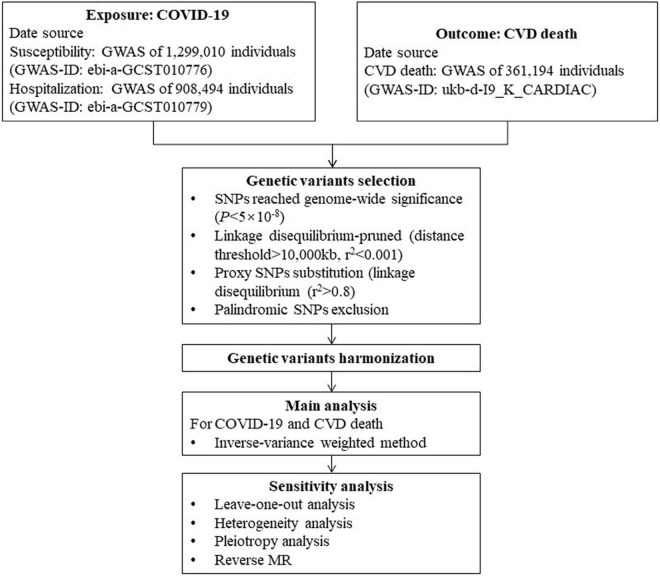
Flow chart of this Mendelian randomization study. COVID-19, Coronavirus disease 2019; GWAS, genome-wide association study; SNP, single nucleotide polymorphism; MR, Mendelian randomization; CVD, cardiovascular disease.

We performed two-sample, bidirectional MR analysis of the relationship between COVID-19 and CVD death through the GWAS database. Univariate MR analysis was performed to assess the impact of COVID-19-related traits on CVD death at the genetic level using COVID-19 susceptibility, hospitalization, and severity as exposure factors and CVD death as the outcome. To test whether there was an inverse causal relationship between the two factors, we conducted reverse univariate MR analysis with CVD death as the exposure factor, along with COVID-19 exposure, hospitalization, and severity as the outcome. The flowchart is presented in Figure 1.

### Data source

Data were obtained from the GWAS database,^1^ from which we extracted COVID-19-related gene data as exposure factors. For the COVID-19 susceptibility analysis data, we included 1,299,010 participants (14,134 patients with confirmed COVID-19 and 1,284,876 population controls; GWAS-ID: ebi-a-GCST010776). For the hospitalized inpatient analysis data, we included 908,494 participants (6,406 COVID-19 inpatients, 902,088 control populations, GWAS-ID: ebi-a-GCST010779). In addition, we extracted genetic data related to CVD death from the GWAS database as an outcome variable (1,597 patients with CVD death, 359,597 patients with non-CVD death; GWAS-ID: ukb-d-I9_K_CARDIAC). Since all data used were publicly available, no additional ethical approval was required. European populations were studied to reduce bias due to race-related confounding factors.

### Instrumental variables

The three conditions that have to be met when using single nucleotide polymorphism (SNP) as IVs in MR analysis are: (1) IVs are associated with exposure factors. (2) IVs can only affect the outcome variable through exposure factors. (3) IVs are not associated with confounders. To remove IVs with chained imbalances, *r*^2^ < 0.001 and kb > 10,000 were set when extracting IVs using the clump_data function. In addition to the above three items, SNPs with genome-wide significance (*P* < 5 × 10^–8^) were selected as IVs. To remove SNPs with linkage disequilibrium (LD), *r*^2^ < 0.001 and kb > 10,000 were set when extracting IVs using the clump_data function. If the selected SNPs were not collected in the outcome GWAS, proxy SNPs in the linkage disequilibrium were used as substitutes. Palindromic SNPs were then removed to ensure that the effect of these SNPs on exposure corresponded to the same allele as the effect on outcome.

### Bidirectional Mendelian randomization and sensitivity analysis

We used the inverse variance weighted (IVW) method to predict the genetic predictive value of the exposure factor for the outcome variable with an effect value of β. For the IVW method, heterogeneity was evaluated using the Q statistic, and a random-effects model was employed to calculate the MR effect size when the Q statistic *P* < 0.05, and vice versa using a fixed effects model. Excessive heterogeneity means that the MR hypothesis may not be valid. We performed the reverse IVW method analysis by pairing the exposure factors with the outcome variables and calculating the Q statistic and *P*-values to assess whether there was a reverse causality.

We performed sensitivity analysis using MR-Egger regression and Leave-one-out analysis, with MR-Egger regression able to detect horizontal pleiotropy. Genetic variation may influence outcome through pathways other than exposure factors, known as horizontal pleiotropy, which violates the MR hypothesis and may lead to bias in causal estimates. To correct this, MR-Egger regression was used to detect horizontal pleiotropy. Leave-one-out analysis re-estimates random effects after removing each SNP individually from the IVW method to assess the stability of the IVW results. If one of the SNPS is invalid, then the distribution of the estimated causal effects in the Leave-one-out analysis may be distorted.

### Statistical analysis

All analyses were performed using the two-sample MR package in R software. If the Q statistic was *P* < 0.05, there was no heterogeneity amongst IVs, but when *P* > 0.05, there was heterogeneity. If *P* > 0.05 in the reverse MR analysis, this indicated that there was no reverse causality between the exposure factors and the outcome variables, so our results prove unidirectional causality. If the MR-Egger regression intercept was not zero and statistically significant (*P* < 0.05), then the IV was considered to have horizontal pleiotropy, and conversely, if *P* > 0.05, then the results were considered not to have horizontal pleiotropy. Furthermore, *P* < 0.05 was considered statistically significant.

## Results

**FIGURE 2:**
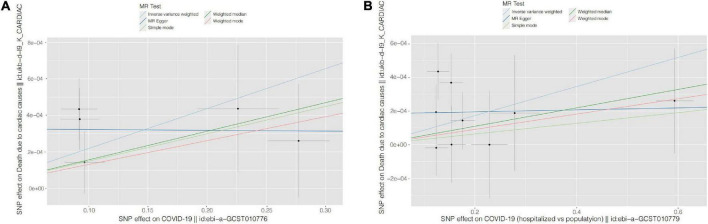
Scatter plots for causal SNP effect of COVID-19 on CVD death. Each black point representing each SNP on the exposure (horizontal-axis) and on the outcome (vertical-axis) is plotted with error bars corresponding to each standard error (SE). The slope of each line corresponds to the combined estimate using each method of the inverse variance weighted (light blue line), the MR-Egger (blue line), the simple mode (light green line), the weighted median (green line), and the weighted mode (pink line). **(A)** COVID-19 susceptibility; **(B)** COVID-19 hospitalization. COVID-19, Coronavirus disease 2019, SNP: single nucleotide polymorphism, MR, Mendelian randomization.

**FIGURE 3:**
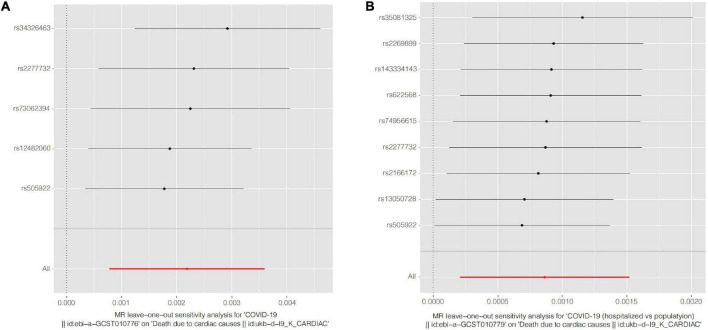
Forest plots of Leave-one-out analyses for causal SNP effect of COVID-19 on CVD death. The error bars indicate the 95% confidence interval (CI). **(A)** COVID-19 susceptibility; **(B)** COVID-19 hospitalization. COVID-19, Coronavirus disease 2019, SNP, single nucleotide polymorphism.

The univariate MR results are shown in Table 1, as well as in Figure 2 (Scatter plots) and Figure 3 (Forest plots).

**TABLE 1:** Univariable MR results of the effect of genetically predicted COVID-19 traits on CVD death risk.

Exposure traits	Outcome traits	Number of SNPs	IVW method		Pleiotropy (MR-Egger method)		Heterogeneity (IVW method)		Reverse MR (IVW method)	
			β	*P* *	Intercept	*P* *	Q	*P* *	β	*P* *
COVID-19 susceptibility	CVD death	5	2.188 × 10^–3^	0.002	0.00033	0.204	4.462	0.347	−0.945	0.968
COVID-19 hospitalization	CVD death	9	8.626 × 10^–4^	0.010	0.00018	0.207	7.968	0.437	−2.74	0.945

MR, Mendelian randomization; COVID-19, Coronavirus disease 2019; SNP, Single nucleotide polymorphism; IVW method, Inverse variance weighted method; CVD, cardiovascular disease.

*Statistical significance was defined as P < 0.05.

For COVID-19 susceptibility, we obtained five common SNPs in tables of both exposure factor and outcome variable after removal of chained unbalanced IVs. The IVW results suggested that infection with COVID-19 may be a causal factor for CVD death compared to population controls (*β* = 2.188 × 10^–3^, *P* = 0.002). We then performed reverse MR analysis, with the results indicating the presence of no detectable reverse causality between COVID-19 and CVD death (*P* = 0.968). The results of the sensitivity analysis showed that the MR-Egger regression analysis suggested the absence of horizontal pleiotropy (*P* = 0.204), and the IVW method revealed the absence of heterogeneity amongst IVs (*P* = 0.347).

For the COVID-19 hospitalization population, we obtained nine common SNPs in tables of both causal factor and outcome variable after removal of chained unbalanced IVs. The IVW results suggested that COVID-19 hospitalization may be a causal factor for CVD death compared to population controls (*β* = 8.626 × 10^–4^, *P* = 0.010). We then performed reverse MR analysis, with the results suggesting that no reverse causality was detected between COVID-19 hospitalization and CVD death (*P* = 0.945). The findings of the sensitivity analysis indicated that MR-Egger regression analysis suggested the absence of horizontal pleiotropy (*P* = 0.207) and IVW method analysis showed the absence of heterogeneity amongst IVs (*P* = 0.437).

## Discussion

Mendelian randomization analysis is effective in avoiding the confounding bias of traditional epidemiological studies, which use genetic variants as IVs and therefore these IVs must follow the laws of Mendelian inheritance. Such IVs are associated with exposure factors and are not directly related to outcome variables. This implies that, if there is a causal relationship between IVs and outcome variables, this relationship accounts for the causal effect of exposure factors on outcome variables and is independent of confounding factors.

A retrospective analysis of 138 COVID-19 patients admitted to the ICU showed that 44.4% had arrhythmias and more comorbidities than non-ICU patients, including high blood pressure (58.3 vs. 21.6%), diabetes (22.2 vs. 5.9%), and cardiovascular disease (25.0 vs. 10.8%) (10). In addition to its potential to increase the risk associated with cardiovascular disease, studies have found that COVID-19 may also increase CVD death. In a retrospective study of 150 patients diagnosed with COVID-19, 68 died, 40% of whom died from cardiovascular disease – significantly higher number than those who recovered (0%) (11). One study included 191 patients, 23.0% (*n* = 44) of whom had heart failure complications, whilst 63.6% (*n* = 28) had a fatal outcome (12). In another meta-analysis of 43 studies involving 3,600 patients, the prevalence of heart failure as a complication was 17.1% in critically ill patients and only 1.9% in non-critically ill patients (13). Wadhera et al. showed an increased risk of CVD death (involving 339,076 patients with CVD deaths) amongst Black, Asian, and Hispanic Americans during the COVID-19 pandemic in the United States in 2020 (14). In contrast, the results of the Shirling Tsai et al. study on female veterans in the United States showed no evidence of increased cardiovascular disease incidence over 60 days in COVID-19-positive individuals compared with COVID-19-negative individuals (involving 8,808 patients with CVD death) (15), and we suggest that this result may be related to follow-up time, gender, age and other factors. Moreover, the COVID-19-positive female patients in this study were younger, aged 48.62 ± 12.66 years, because the risk of cardiovascular events is only increased in postmenopausal women (16). Whilst the above studies have found a correlation between COVID-19 and CVD death from clinical data, our study takes the genetic approach further.

The results of Anurag Verma et al. showed that the rs505922 SNP in the ABO blood group gene was significantly associated with COVID-19 severity and hospitalization (17). Similarly, our findings found an association between both COVID-19 severity and hospitalization and CVD death, with rs505922. Moreover, rs505922 was found to be a coronary-prone locus and was associated with the occurrence of myocardial infarction (18).

A noteworthy study by Anurag Verma and Matteo D’Antonio showed that rs2277732 was associated with hospitalization and severity of COVID-19, respectively (19, 20), whilst the results of the present study similarly found that rs2277732 was associated with both hospitalization and severity of COVID-19 and CVD death, but there exist no studies involving the association of rs2277732 with CVD death. These results suggest that the above-mentioned COVID-19-related SNPs may indirectly increase CVD death through other factors such as blood type, but this is only an inference and needs to be verified by further studies.

The exact underlying mechanism of increased CVD death due to COVID-19 is not well understood. The excessive production of cytokines and chemokines such as interleukin-6, 12, 1β and interferon-γ by the organism in patients with severe COVID-19 may contribute to cardiac injury (21). Another study showed that tissues expressing ACE2 > 1% are at high risk of SARS-CoV-2 virus infection, and up to 7.5% of cardiac tissues express ACE2, which may lead to severe myocardial injury and even progression to CVD death after SARS-CoV-2 infection (22). Acute phase myocardial injury may be due to the direct effect of cytopathic lesions, leading to myocardial ischemia, gap junctional dysfunction, and altered ion channels giving rise to cardiac electrical remodeling and thus arrhythmogenesis (23).

Our study also has some limitations. First, the amount of data related to COVID-19 severity and CVD death in the GWAS database is too small to perform data analysis in this population. Second, since the results of this study are the product of statistical analysis, more clinical research studies are needed to support our findings and to elucidate the possible specific mechanisms by which COVID-19 affects CVD death mechanisms.

## Conclusion

Our study assessed the random effects of COVID-19 susceptibility, hospitalization, and severity on CVD death *via* a two-sample MR methodology using a collection of data information from the GWASs database. Our bidirectional MR analysis showed a causal relationship between COVID-19 susceptibility and hospitalization associated with an increased risk of CVD death, suggesting that COVID-19 is a risk factor for CVD death. However, as the underlying mechanisms are still unclear, further studies are necessary to validate our findings and investigate those underlying mechanisms.

## Data availability statement

The original contributions presented in this study are included in the article/supplementary material, further inquiries can be directed to the corresponding author.

## Author contributions

R-ZS conceptualized the study and drafted the initial manuscript. R-ZS, J-PM, and X-YG analyzed and explained the data, have collected the data, conceptualized and designed the study, and critically reviewed and revised the manuscript. All authors contributed to the article and approved the final version of the manuscript.

## Footnotes

1
https://gwas.mrcieu.ac.uk/
